# Supplementary material for: Empirical methods for controlling false positives and estimating confidence in ChIP-Seq peaks
Source: BMC Bioinformatics. 2008 Dec 5;9:523. doi: 10.1186/1471-2105-9-523 (PMC2628906; doi:10.1186/1471-2105-9-523)
Supplement: Additional File 1 — A variety of html documents from the USeq web site detailing the available applications, their best usage, output file type descriptions, command line menus, etc. [file 1471-2105-9-523-S1.zip › USeqUserGuides/JavaDoc.zip!/JavaDoc/bioroot/ContactBean.html]

ContactBean


|  |  |  |
| --- | --- | --- |
| **Overview** \| **Package** \| **Class** \| **Use** \| **Tree** \| **Deprecated** \| **Index** \| **Help** | |  |
| **PREV CLASS**   **NEXT CLASS** | **FRAMES**    **NO FRAMES**     **All Classes** |  |
| SUMMARY: NESTED \| FIELD \| CONSTR \| METHOD | DETAIL: FIELD \| CONSTR \| METHOD |  |


---


## bioroot Class ContactBean

```
java.lang.Object
  bioroot.ContactBean
```

---

``` public class ContactBean extends java.lang.Object ```

**Author:**
:   nix
    Holds info related to making an email contact with regards to a reagent(s)

---

**Constructor Summary**

`ContactBean()`


| **Method Summary** | |
| --- | --- |
| `java.lang.String` | `getContactEmail()` |
| `int` | `getId()` |
| `java.lang.String` | `getLabGroupName()` |
| `java.lang.String` | `getMessage()` |
| `java.lang.String` | `getMessages()` |
| `java.lang.String` | `getOwnerName()` |
| `java.lang.String` | `getReagentNames()` |
| `java.lang.String` | `getRequesterEmail()` |
| `java.lang.String` | `getType()` |
| `void` | `loadContactInfo(DBUtil bioRoot)` |
| `void` | `setContactEmail(java.lang.String contactEmail)` |
| `void` | `setId(int id)` |
| `void` | `setInitialMessage()` |
| `void` | `setLabGroupName(java.lang.String labGroup)` |
| `void` | `setMessage(java.lang.String message)` |
| `void` | `setMessages(java.lang.String messages)` |
| `void` | `setOwnerName(java.lang.String name)` |
| `void` | `setReagentNames(java.lang.String reagentNames)` |
| `void` | `setRequesterEmail(java.lang.String requesterEmail)` |
| `void` | `setType(java.lang.String type)` |

**Methods inherited from class java.lang.Object**

`equals, getClass, hashCode, notify, notifyAll, toString, wait, wait, wait`

**Constructor Detail**

### ContactBean

```
public ContactBean()
```


**Method Detail**

### setInitialMessage

```
public void setInitialMessage()
```

---


### loadContactInfo

```
public void loadContactInfo(DBUtil bioRoot)
```

---


### getId

```
public int getId()
```

---


### setId

```
public void setId(int id)
```

---


### getMessage

```
public java.lang.String getMessage()
```

---


### setMessage

```
public void setMessage(java.lang.String message)
```

---


### getType

```
public java.lang.String getType()
```

---


### setType

```
public void setType(java.lang.String type)
```

---


### getLabGroupName

```
public java.lang.String getLabGroupName()
```

---


### setLabGroupName

```
public void setLabGroupName(java.lang.String labGroup)
```

---


### getOwnerName

```
public java.lang.String getOwnerName()
```

---


### setOwnerName

```
public void setOwnerName(java.lang.String name)
```

---


### getContactEmail

```
public java.lang.String getContactEmail()
```

---


### setContactEmail

```
public void setContactEmail(java.lang.String contactEmail)
```

---


### getMessages

```
public java.lang.String getMessages()
```

---


### setMessages

```
public void setMessages(java.lang.String messages)
```

---


### getReagentNames

```
public java.lang.String getReagentNames()
```

---


### setReagentNames

```
public void setReagentNames(java.lang.String reagentNames)
```

---


### getRequesterEmail

```
public java.lang.String getRequesterEmail()
```

---


### setRequesterEmail

```
public void setRequesterEmail(java.lang.String requesterEmail)
```


---


|  |  |  |
| --- | --- | --- |
| **Overview** \| **Package** \| **Class** \| **Use** \| **Tree** \| **Deprecated** \| **Index** \| **Help** | |  |
| **PREV CLASS**   **NEXT CLASS** | **FRAMES**    **NO FRAMES**     **All Classes** |  |
| SUMMARY: NESTED \| FIELD \| CONSTR \| METHOD | DETAIL: FIELD \| CONSTR \| METHOD |  |


---
